# Treatment of Anterior Dislocation of the Sacroiliac Joint *via* the Lateral‐rectus Approach: Surgical Techniques and Preliminary Outcomes

**DOI:** 10.1111/os.13794

**Published:** 2023-07-10

**Authors:** Shicai Fan, Sheqiang Chen, Qiguang Mai, Tao Li, Yuhui Chen, Zhenhua Zhu, Hua Wang, Cheng Yang, Jianwen Liao, Ruipeng Zhang, Yingze Zhang

**Affiliations:** ^1^ Department of Traumatic Surgery, Center for Orthopaedic Surgery Third Affiliated Hospital of Southern Medical University Guangzhou China; ^2^ Trauma Emergency Center Third Hospital of Hebei Medical University Shijiazhuang China

**Keywords:** Anterior dislocation, Lateral‐rectus approach, Lumbosacral plexus, Pelvic fractures, Sacroiliac joint

## Abstract

**Objective:**

Anterior dislocation of the sacroiliac joint (ADSIJ) is caused by strong violence, and because of its low morbidity, there are no standardized diagnostic and therapeutical guidelines at this moment. This study aims to explore the surgical techniques and preliminary outcomes of the lateral‐rectus approach (LRA) for treating ADSIJ.

**Methods:**

A retrospective study was conducted of 15 patients with ADSIJ from January 2016 to January 2021. The patients' age ranged from 1.8 years old to 57 years old (37 ± 18 years old). All patients underwent open reduction and internal fixation (ORIF) through the LRA. Eight patients were combined with lumbosacral plexus injury and underwent neurolysis during operation. Patients' fracture type, mechanism of injury, associated injuries, operation time and intraoperative bleeding volume were accessed by reviewing medical history. Quality of fracture reduction was evaluated with the Matta score. At 1‐year follow‐up, the functional rehabilitation was evaluated by the Majeed rehabilitation criteria. For those with lumbosacral plexus injury, the neuromotor function was evaluated using muscle strength grading proposed by the British Medical Research Council (BMRC) and recovery was recorded.

**Results:**

All 15 patients underwent the operation successfully. The surgical time ranged from 70 to 220 min (126 ± 42 min), and the intraoperative blood loss ranged from 180 to 2000 mL (816 ± 560 mL). Eighty percent of the cohort (12/15) were rated as excellent and good in the Matta score for fracture reduction quality after operation without surgical incision‐related complications. At 1‐year follow‐up, the overall excellent and good rate was 73.3% (11/15) according to the Majeed criteria, the neuromotor function recovered completely in six cases and partially in two cases according to the BMRC muscle strength grading, and the recovery of sensory function was evaluated as excellent in six cases, good in one case and poor in one case, with an overall excellent and good rate of 87.5%.

**Conclusion:**

The LRA can well expose the surrounding structures of the sacroiliac joint from the front, which helps surgeons reduce and fix the anterior dislocation of the sacroiliac joint under direct vision and effectively decompress the entrapment of the lumbosacral plexus to achieve better clinical efficacy.

## Graphical abstract

Fifteen patients suffered anterior dislocation of sacroiliac joint and eight of them had lumbosacral plexus injury. They were treated with the lateral rectus approach (LRA) and had good clinical efficacy.

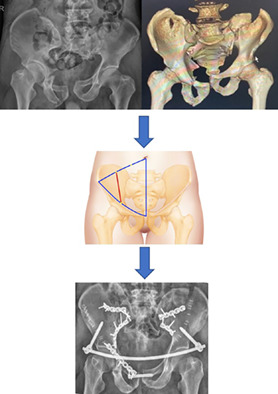

## Introduction

**Fig. 1:**
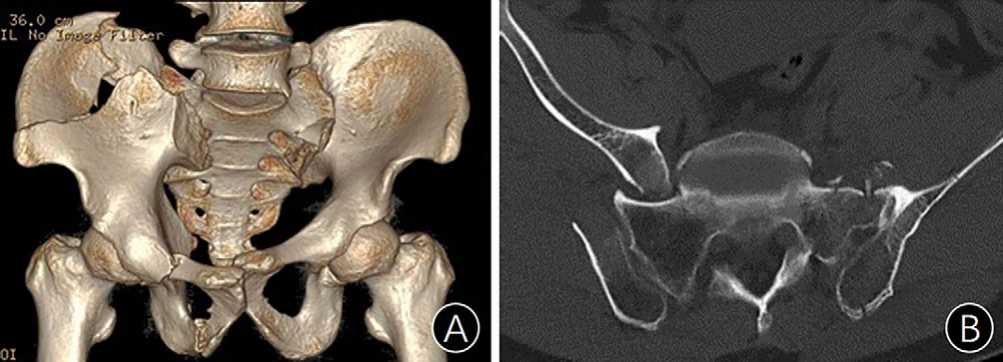
(A) Iliac auricular surface is displaced to the front of the sacral wing; (B) The ilium forms an interlock with the sacral wing.

Anterior dislocation of the sacroiliac joint (ADSIJ) refers to the displacement of the iliac partially or entirely shifting to the front of the sacrum after pelvic injury, forming an interlock (Fig. 1). ADSIJ is so rare in clinical practice that only a few studies with small number of cases on such injury have been reported so far.^1^, ^2^, ^3^, ^4^, ^5^ Lewis and Arnold^6^ first described ADSIJ in their case report of two patients in 1976.

The sacroiliac joint belongs to the amphiarthrosis joint, which is composed of the articular surface of the sacrum and the iliac bone and the ligament complex. The sacral surface is slightly depressed while the iliac surface is relatively convex, which has an interlocking effect to stabilize the joint like an enarthrosis. The bone and strong ligament complex jointly resist the deforming force. The occurrence of sacroiliac joint dislocation indicates that patients suffer from lateral extrusion or rotation force of injury. Since the sacrum is wide in the front and narrow in the back, sacroiliac injury is more often manifested as posterior or vertical dislocation.^7^ The reason for the ADSIJ is that once the iliac bone suffers powerful force that leads to its anterior dislocation,^8^, ^9^ it may form an interlock and hinder reduction during surgery.

The dislocation of the sacroiliac joint can be classified into two subtypes: A, the posterior dislocation of the sacroiliac joint; and B, the anterior dislocation of the sacroiliac joint. The type B can be further subdivided into two subgroups, including: (i) anterior dislocation of the sacroiliac joint combined with no fracture or stable fractures of hip bones; and (ii) anterior dislocation of the sacroiliac joint combined with the unstable fractures of hip bones and/or contralateral SI joint injury.^10^

After the occurrence of ADSIJ, the posterior pelvic ring is severely damaged, combining with anterior pelvic ring injury, which makes the whole ring vertically and rotationally unstable.^11^, ^12^ As the lumbosacral plexus (LSP) and other important venous plexus are in front of the sacrum, they can be easily damaged in the case of ADSIJ. It is reported in the literature that the incidence of pelvic fracture with nerve injury is 0.75% ~ 15%,^13^, ^14^, ^15^ while the incidence of severe sacroiliac joint fractures with lumbosacral plexus injury is as high as 25% ~ 66%.^16^ It is necessary to reconstruct the pelvic ring and explore and release the nerve in terms of ADSIJ. The surgical methods include external fixation, closed reduction, anterior or posterior open reduction combine with internal fixation. The low morbidity of ADSIJ leads to its controversial therapeutic regimen.^17^ For partial ADSIJ injury, patients will be recommended external fixation if their vital signs are unstable or they suffer open fractures, and internal fixation will be performed timely once they are stable. The available internal fixation includes anterior plates, sacroiliac screws, or anterior plates combined with sacroiliac screws, in which sacroiliac screws associated with leverage or double plates fixation are ideal treatments.^1^, ^10^

The most widely used anterior approaches for exposing sacroiliac joint include the iliac fossa approach, the para‐rectus approach, and the lateral‐rectus approach (LRA). However, the iliac fossa approach may cause the injury of vascular bundle and lateral femoral cutaneous nerve (LFCN).^18^, ^19^ The para‐rectus approach has the risk of perforating the peritoneum, dissection of the artery, thromboembolism, and lesion of LFCN or femoral nerve.^20^, ^21^ Among them, the LRA is an anterior approach that can expose the pelvis from the symphysis pubis to the sacroiliac joint, through which surgeons can decompress the lumbosacral plexus nerve and achieve satisfactory fracture reduction of both anterior and posterior pelvic ring. It is a better option for patients suffering ADSIJ.

Our team has been using the LRA to treat patients with ADSIJ since 2016, which has achieved good clinical efficacy. This study retrospectively analyzed the clinical data of patients who suffered ADSIJ injury and underwent surgery *via* the LRA from January 2016 to January 2021 and evaluated their surgical methods and treatment effects.

The purposes of this study were to: (i) analyze the characteristics of ADSIJ injury; and (ii) evaluate the surgical techniques and preliminary outcomes of the LRA for treating ADSIJ injury.

## Materials and Methods

### Patients

This study has been approved by the ethics committee (ethical approval number: 201508006) and informed consent was obtained from all included participants.

A total of 15 ADSIJ patients with surgical treatment were included in this study. There were seven males and eight females. The average age ranged from 1.8 years old to 57 years old (mean 37.0 ± 18.1 years old). All 15 patients were associated with anterior pelvic ring fractures. Among them, there were six patients combined with contralateral posterior pelvic ring injury, eight patients with ipsilateral lumbosacral plexus injury, seven patients with pelvic organ injuries, and two patients with ipsilateral acetabular fracture. The time from injury to surgery ranged from 7 to 145 days (mean 19.8 ± 35.0 days). The causes for the injury can be classified as falling from height (four cases), traffic accidents (six cases) and crush injuries (five cases). The classification of the ADSIJ were as followed: seven cases of type B.a and eight cases of type B.b. All these data, including the amount of intraoperative bleeding and operation time, are shown in Table 1.

**TABLE 1:** Patients' general data

Sequence	Gender	Age	Injury pattern	Dislocation site	Pelvic ring injury	Associated injuries	Lumbosacral plexus injury	Surgery time (min)	Blood Loss (mL)	Time before surgery (d)
1	Male	51	Crushing	Left	Pubic symphysis dislocation	Intestine Injury	No	70	400	8
2	Male	37	Traffic accident	Right	Ipsilateral acetabular and bilateral pubic ramus fracture	Urethral Rupture	Yes	170	800	10
3	Male	47	Falling	Right	Contralateral iliac fracture and pubic symphysis dislocation	Urethral rupture	Yes	165	1200	15
4	Female	47	Falling	Right	Ipsilateral acetabular and bilateral pubic ramus fracture	Numerus fracture	No	220	2000	145
5	Male	55	Crushing	Left	Bilateral pubic ramus fracture	Urethral rupture	Yes	85	680	9
6	Female	1.8	Crushing	Right	Contralateral iliac fracture and pubic symphysis dislocation	Perineum injury	No	115	250	25
7	Male	48	Crushing	Right	Bilateral pubic ramus fracture	Intestine injury	Yes	80	500	9
8	Male	57	Traffic accident	Right	Ipsilateral pubic ramus fracture	Bladder injury	Yes	90	460	7
9	Female	24	Traffic accident	Left	Ipsilateral pubic ramus fracture	No	Yes	135	660	7
10	Female	2	Crushing	Left	Ipsilateral pubic ramus fracture	Perineum injury	No	160	180	10
11	Female	47	Falling	Right	Contralateral iliac fracture and bilateral pubic ramus fracture	Limbs fracture	No	135	1440	13
12	Female	39	Traffic accident	Right	Contralateral pubic ramus and sacral fracture	Intestine injury	Yes	125	1750	15
13	Female	25	Falling	Left	Contralateral sacral fracture and pubic symphysis dislocation	Limbs fracture	No	150	1100	8
14	Male	22	Traffic accident	Right	Ipsilateral pubic ramus fracture	No	No	80	350	7
15	Female	52	Traffic accident	Left	Contralateral pubic ramus and sacral fracture	No	Yes	115	470	9

### Inclusion and Exclusion Criteria

Inclusion criteria included: (i) pelvic ring injury caused by trauma; (ii) anterior sacroiliac joint dislocation; (iii) patients were treated with surgery; and (iv) surgical field was exposed through the LRA.

Exclusion criteria included: (i) sacroiliac joint separation or posterior dislocation; (ii) no obvious fracture displacement; and (iii) incomplete follow‐up or loss to follow‐up.

### Perioperative Management

Related routine preoperative examinations were performed for all patients after admission. The pelvic CT scan (Siemens, Munich, Germany) was performed on all patients. And the data was inputted into Mimics software (Materialise, Leuven, Belgium) for three‐dimension (3D) reconstruction. An equal‐large 3D model was manufactured by a 3D printer (Stratasys Dimension 1200es, Migdal HaEmek, Israel) to help surgeons clearly understand the type of fracture and the anterior dislocation of the sacroiliac joint. Patients with LSP injuries underwent LSP MRI scan reconstruction to determine the location of nerve injury, which can assist surgeons to formulate a detailed preoperative plan of neurolysis. The color Doppler ultrasonography of the lower extremity was performed 1 day before surgery to rule out deep vein thrombosis of the lower extremity. A broad‐spectrum antibiotic was given intravenously 30 min before surgery. And the antibiotic was given again with over 3 h of operation time or over 1000 mL of intraoperative blood loss. The blood was prepared, and intraoperative autologous blood transfusion was performed.

### Surgical Technique

Patients were placed in the supine position after general anesthesia with endotracheal intubation. And sterile bandage of the affected lower extremity was performed for intraoperative traction.

#### Approach

**Fig. 2:**
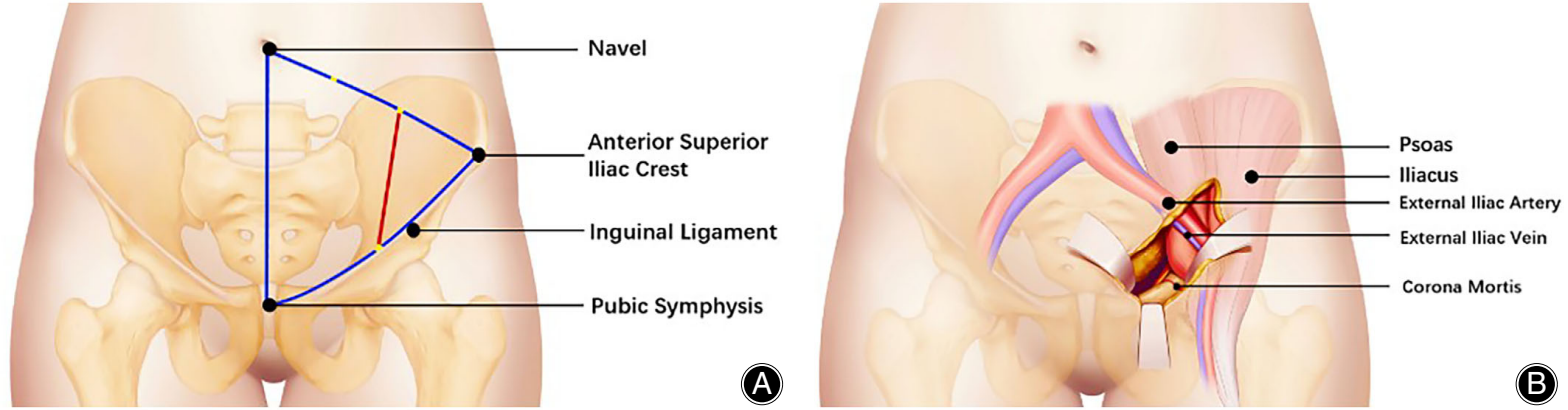
(A) The incision of the LRA; (B) The medial window of the LRA.

The standard incision of the LRA^22^, ^23^, ^24^, ^25^ starts at the outer one‐third of the link from the umbilicus to the anterior superior iliac spine and ends at the midpoint of the inguinal ligament, with a length of roughly 8 cm (Fig. 2A). The extraperitoneal space is exposed after subcutaneous dissection and incision of the obliquus externus abdominis, obliquus internus abdominis, transverse abdominis, and ends at the medial margin of the superficial inguinal ring. There are three windows of the LRA: the medial window (Fig. 2B) exposes the anterior pelvic ring; the middle window exposes the structures around the sacroiliac joint; and the presacral window (or lateral window) exposes the front of the sacrum and the S1 nerve foramen if patients suffer S1 nerve injuries.

#### Exposure, Reduction, and Fixation

After separating the peritoneum and the muscle bluntly, the medial window was opened, and the anterior pelvic ring was exposed. For the patients companied with S1 nerve injuries, the presacral window was made to expose the front of the sacrum and sacral foramen. The middle window could reveal from the greater sciatic foramen to the auricular surface of the ilium that could be seen because of ADSIJ injury. After exposing the middle window through the space between the iliopsoas muscle and external iliac vessels, it was exposed upward along the sciatic foramen to the iliac auricular surface of the sacroiliac joint with anterior dislocation. The soft tissue around the articular surface had been carefully separated before the Schanz nail was inserted into anterior superior iliac crest, for the lumbosacral plexus and iliac vessels might be attached to the surface. After the Schanz nails were set, surgeons could loosen the sacroiliac joint by spinning the ilium outward and forward carefully in case of hurting the lumbosacral plexus and reduce the ilium while pulling the affected lower limbs. If the reduction were difficult to achieve, surgeons could try spinning and pulling the Schanz nails outward to enlarge the gap between the ilium and sacrum, insert the periosteal detacher into it simultaneously to release the blockage, and then reduce the ilium by traction. Fixation was completed with two plates placed supra‐pectineally and infra‐pectineally across the sacroiliac joint, or with a reconstruction plate placed in front of the sacroiliac joint and an S1 sacroiliac screw. Reduction of old ADSIJ injury (more than 3 weeks) was relatively more difficult than that of the fresh one (less than 3 weeks), which required ilium osteotomy and sacroiliac joint bone grafting after eliminating the tissue stuck in the joint to avoid aggravating the nerve damage by repeatedly pulling the iliac bone back and forth during the operation. In our cohort of 15 patients, three of them went through iliac articular surface osteotomy.

The reduction and fixation of the ADISJ can be performed with a 3.5 or 4.5 mm reconstructed plate or an anterior sacroiliac anatomic plate. One screw is drilled through the sacrum, parallel to the sacroiliac articular surface. The screw direction is consistent with the direction of the longitudinal axis of the iliac bone, which can make screws reach enough depth within the ilium to achieve structural stability. Full‐threaded cancellous bone screws, with a length between 30 and 40 mm, are common choices for fixing the plate on the iliac wing. After the fixation of the first plate, add the other plate to the ilium. The two plates can be placed parallel or crossed at an angle of 60° ~ 90°. Locking plates are an ideal alternative for osteoporotic patients to increase the strength of fixation.

### Postoperative Management

After the operation, the drainage tube was routinely placed, and it was pulled out when the drainage volume was less than 50 mL. Routine anticoagulation was started 6 h after operation to prevent deep vein thrombosis of lower extremities. Within 24 h after operation, patients were instructed to perform isotonic and isometric contractions of lower extremity muscles, and actively or passively move hip and knee joints. Those with LSP injury were given corresponding nerve stimulation treatment. The rehabilitation and exercising time of the patients hinged on the severity of the fracture, reduction and fixation, combined injuries, etc. Generally, the patients could walk with partial weight‐bearing 4–6 weeks after the operation. At 1, 3, 6 and 12 months after the operation, the pelvic X‐rays and CT scans were performed to evaluate the fracture reduction quality, the position of the internal fixator, the healing of fractures and the data of limbs functional recovery were recorded. Patients with LSP injuries were treated with acupuncture and moxibustion 1 week after operation to promote the recovery of nerve function.

### Efficacy Evaluation

#### Outcome Measurement

The operation time, intraoperative blood loss for the exposure, reduction, and fixation of ADSIJ were recorded. Pelvis X‐rays and CT scans were performed postoperatively for all patients. Fracture healing was assessed based on the follow‐up imaging results and physical examination.

#### Imaging Results and Physical Examination

Fracture healing status was evaluated according to imaging results and physical examination. As long as the X‐ray showed blurred fracture lines and continuous callus formation, and the patients showed no pain at the sacroiliac joint during walking, it was evaluated as clinical fracture healing.

#### Matta Criteria

The postoperative pelvic fracture reduction quality was measured using the Matta criteria^26^ according to the maximal shift measured on the anterior–posterior (AP), inlet and outlet views of the pelvis. The results of displacement were classified into four levels: excellent (4 mm or less), good (5–10 mm), fair (10–20 mm) and poor (more than 20 mm).

#### Majeed Functional Score

The Majeed functional score^27^ was used to evaluate postoperative function: patients were scored in working (20 points), pain (30 points), sitting (10 points), standing (36 points) and sexual intercourse (4 points), where a total score was 100 points. Among them, if the patients had no sexual intercourse or work before the injury, their revelent scores would be full marks. The scores can be divided into four levels: excellent (85 points or more), good (70 to 84 points), fair (55 to 69 points), and poor (less than 55 points).

#### Muscle Strength Grading and Sensory Function Evaluation

According to the British Medical Research Council (BMRC) nerve injury classification,^28^ the recovery of neuromotor function before and after surgery was evaluated as follows: M0 means no muscle contraction; M1 means contractile recovery of proximal muscle; M2 means contractile recovery of proximal and distal muscles; M3 means that major muscles are powerful enough to countermine gravity; M4 means all movements and coordination can be done actively; and M5 means completely normal function. Evaluation of sensory function^29^ after peripheral nerve injury was as follow: S0 represents no sensory recovery; S1 represents a deep sensory recovery of the skin in the region of innervation; S2 represents a partial recovery of superficial sensibility and tactile sensation in the region of innervation; S3 represents a recovery of skin pain and tactile sensation and disappearance of sensory allergy; and S4 represents a complete recovery of sensation. The results were divided into 4 levels: excellent (S4), good (S3), fair (S2), and poor (S0 ~ S1).

### Statistical Analysis

All data in the study were analyzed using SPSS 26.0 (SPSS Inc., Chicago, IL, USA). The measurement data was accessed by the Shapiro–Wilk test to determine whether the data is normally distributed. Normally distributed data are presented in the form of mean ± standard deviation.

## Results

### General Outcomes

The average operation time of the LRA in treating ADSIJ ranged from 70 to 220 min (mean 126 ± 42 min), and the average intraoperative blood loss ranged from 180 to 2000 mL (mean 816 ± 560 mL), which are less than those of the LRA in treating pelvic fracture with lumbosacral plexus injury reported in the literature.^30^ These data are summarized in Table 1.

### Fracture Reduction

In this group, 15 ADSIJ patients achieved clinical healing, and such complications as displacement of fracture, delayed union or nonunion of fractures, incision infection and other clinical complications did not occur. Postoperative reduction quality of ADSIJ using the Matta criteria was as follows: excellent in eight cases, good in four cases, and fair in three cases, with a total excellent and good rate of 80%.

### Recovery of Function

The follow‐up time was 12 ~ 36 months. According to the Majeed functional score, there were six cases of excellent, five of good, three of fair, and one of poor at 1‐year follow‐up after operation. The overall excellent and good rate was 73.3%. According to BMRC, the recovery of lumbosacral plexus function in eight patients with lumbosacral plexus injury after operation was evaluated as follows: six cases of M5, one case of M4, and one case of M2 in motor function recovery. The recovery of sensory function was evaluated as excellent in six cases, good in one case, and poor in one case, with an overall excellent and good rate of 87.5%.

### Typical Case

**Fig. 3:**
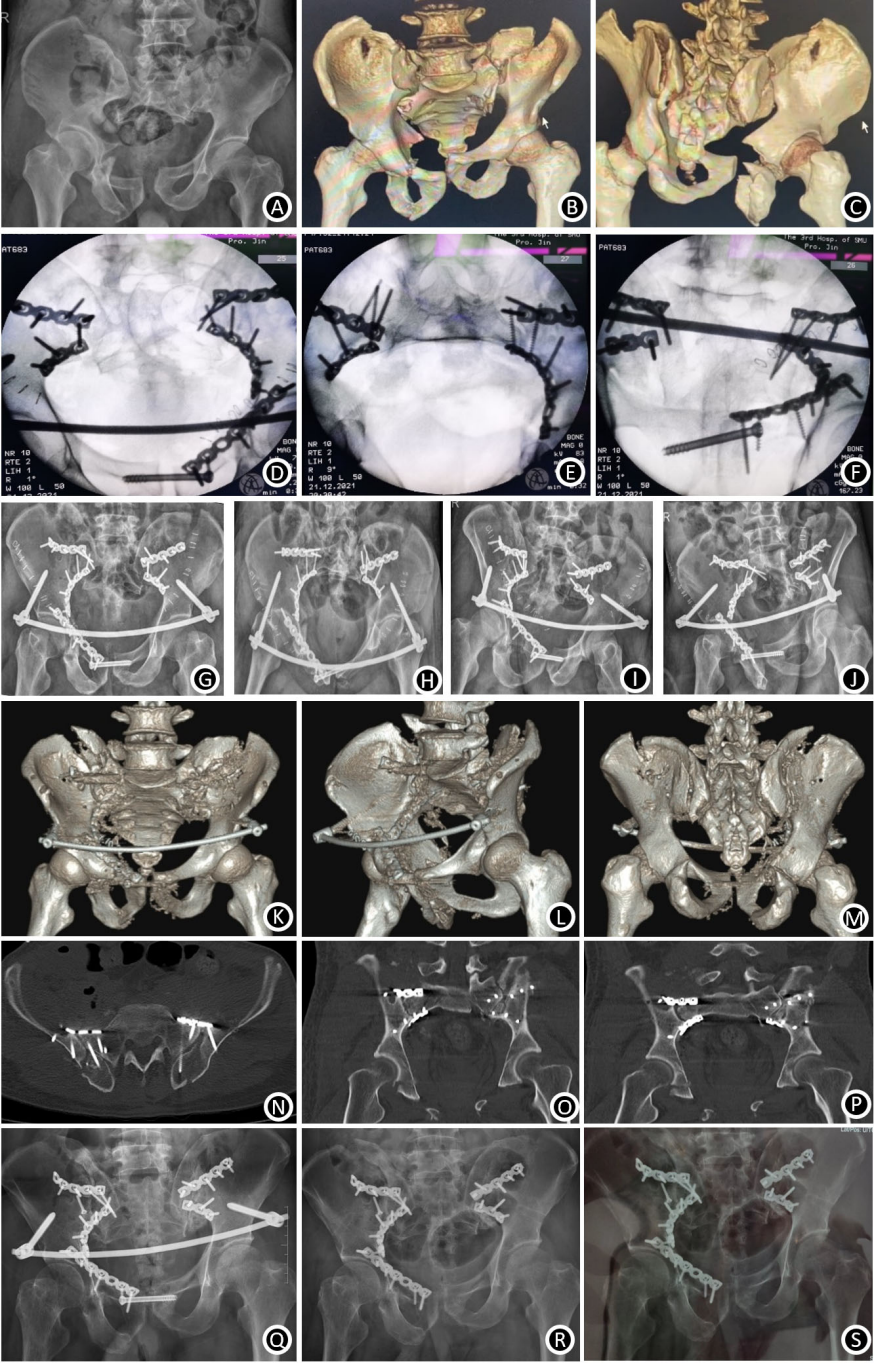
(A–C) Preoperative AP view and 3‐D reconstruction of CT data showed bilateral crescent‐shaped fractures of the posterior pelvic ring, anterior dislocation of the right sacroiliac joint, fractures of the superior and inferior pubic rami, and separation of the pubic symphysis; (D–F) AP, inlet and outlet views during operation: reduced the dislocated ilium by placing a plate on the sacroiliac joint and another plate on the arcuate line, and the anterior pelvic ring was fixed by a lag screw, an anterior column reconstructive plate and an internal fixator (INFIX); (G–M) Postoperative AP, inlet, obturator oblique, iliac oblique views, 3‐D reconstruction of CT data: ideal reduction and internal fixation was achieved; (N–P) Postoperative CT scan: the sacroiliac joint was well reduced and the angulated plates provided biplane stability for the joint; (Q) Postoperative AP view at 4‐month follow‐up before removing the INFIX frame; (R) Postoperative AP view at 4‐month follow‐up after removing the INFIX frame; (S) Postoperative AP view at 1‐year follow‐up: no fragments dislocation or loosening of internal fixation.

A 47‐year‐old male, who suffered a pelvic fracture with complete injury to the right lumbosacral plexus after a fall from height, underwent fracture reduction and fixation *via* the bilateral LRA 15 days after the injury (Fig. 3). At the 1‐year follow‐up after the surgery, the patient could walk normally with well recovery of the function of the right lumbosacral plexus.

## Discussion

In our study, the LRA can well expose the sacroiliac joint, thus providing enough space for ideal reduction and firm fixation of the ADSIJ injury. As for those with lumbosacral plexus injury, the LRA is capable of releasing the nerve and promoting fast rehabilitation.

### Mechanisms of Injury

**Fig. 4:**
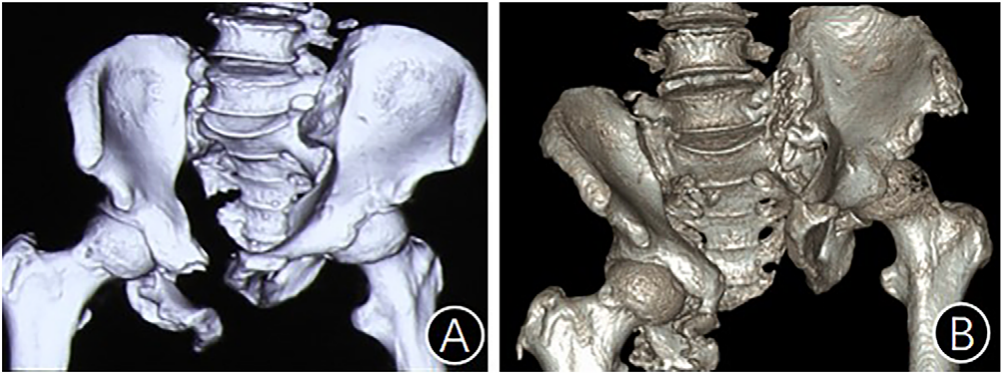
ADSIJ injuries: severe instability of the posterior pelvic ring and obvious external rotation deformity of the iliac wings. (A) The patient suffered external rotation and anterior dislocation of the left sacroiliac joint after injury, compression of the right sacral wing and internal rotation of the right iliac wing; (B) After 1 year of nonoperative treatment, the fracture healed but the pelvic ring was severely deformed, and the patient could not sit, stand or walk due to the deformity.

**Fig. 5:**
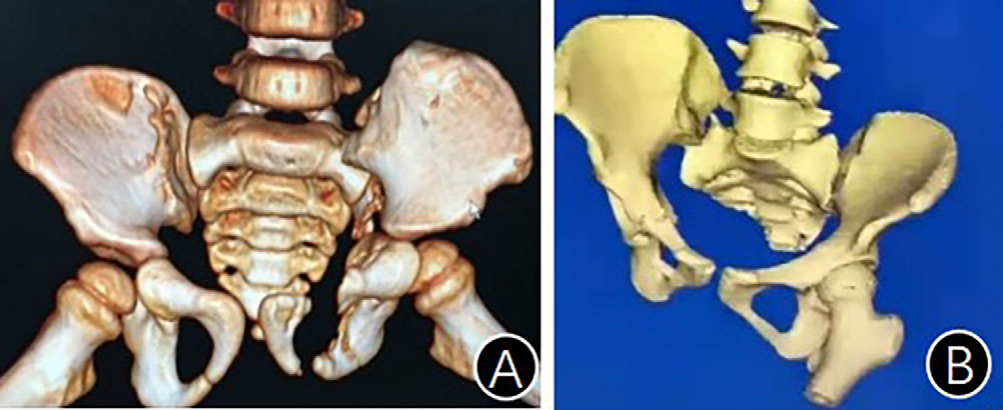
(A, B) ADSIJ in Children is often manifested by total anterior dislocation, with the entire auricular surface of ilium dislocated to the anterior part of the sacral wing.

**Fig. 6:**
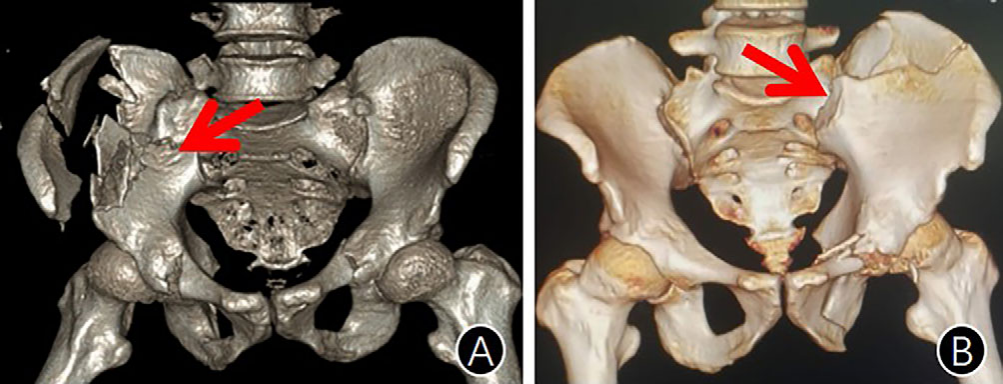
(A, B) ADSIJ in adults is often manifested by peri‐sacroiliac fracture and dislocation.

ADSIJ is common in high‐energy injuries resulting in sacroiliac complex rupture or iliac fractures, as well as the disruption of the integrity of the anterior pelvic ring. The severe instability of the posterior pelvic ring and obvious internal rotation deformity of the iliac wings hinder limb function recovery (Fig. 4). Children suffer higher morbidity than adults and most of them manifest as complete anterior dislocation of the sacroiliac joint (Fig. 5), which may be related to the weaker strength of the ligament around the sacroiliac joint, insufficient bone calcium deposition, and good bone flexibility.^10^ While adults often present as fractures or partially dislocation of the sacroiliac joint (Fig. 6). Two children in this group had complete anterior dislocation of the sacroiliac joint. The literature^1^ reports that ADSIJ is caused by the great lateral compression on the injured side of the pelvic ring, resulting in the rupture of the sacroiliac complex or/and crescent fracture of the pelvis, partial or complete separation of the sacroiliac joint anterior to the front of the sacral alar. Therefore, the anterior dislocation of the sacroiliac joint should be classified as the LC type in the Young–Burgess classification. Meanwhile, pelvic fractures with ADSIJ should be classified as type C fractures in Tile or AO/OTA classifications due to both vertical and rotational instability, whereas type C fractures cannot be caused by lateral compression. Therefore, the key to the mechanism of injury for pelvic fractures with ADSIJ is sufficiently strong violence, which can be caused by lateral compression, anterior–posterior compression, vertical shearing, or the existence of combined or secondary violence. Among the injury pattern of 15 patients in the cohort, six were traffic injuries, four were fall from heights, and five were crush injuries, all of which were combined with the anterior pelvic ring injury. Among them, there were six cases of contralateral posterior pelvic ring injury, two cases of ipsilateral acetabular fracture, seven cases of injury to internal organs such as the bladder and rectum, and eight cases of ipsilateral lumbosacral plexus injury. From the above, ADSIJ injuries are mostly multiple injuries caused by powerful violence.

### Surgical Method Selection

Due to the severe deformity and instability of the pelvic ring and limbs function restriction after ADSIJ injury, early surgical treatment ought to be taken to restore the pelvic ring structure and regain its stability. As closed reduction is not an ideal treatment for ADSIJ, early open reduction is recommended to avoid lumbosacral plexus injury. The key is to fully expose the anatomical structures around the dislocated sacroiliac joint, relieve the stuck soft tissue, provide enough operative space for reduction, and decompress and release the lumbosacral plexus. Both anterior approaches and posterior approaches have been reported previously.^31^, ^32^ Most surgeons chose the ilioinguinal approach^33^ to reduce and fix by full exposure of the sacroiliac joint through its lateral window. However, it cannot sufficiently expose the anteromedial tissues of the sacroiliac joint, such as presacral vessels and lumbosacral plexus, and has a risk of damaging vascular nerves. The posterior iliac crest approach was also a favorite approach nowadays, through which the posterior sacroiliac joint can be directly exposed, decompressed, and reduced. The surgical exposure is relatively safe. For old dislocations, the posterior approach can simultaneously release the sacrospinous ligament and the sacrotuberous ligament, making it more suitable for complete anterior dislocation of the sacroiliac joint, whose clinical efficacy is better than that of the anterior approach. However, it may damage the anterior sacral vessels and nerves without their exposure during the operation and that is why it is not suitable for patients with large iliac bone fragments remaining in the rear part of the auricular surface of the sacrum (such as crescent type I fracture of the pelvis with the anterior dislocation). In addition, ADSIJ injuries are often associated with anterior pelvic ring fracture or lumbosacral plexus injury. The posterior approach cannot release the nerves, and the anterior approach should also be added for the reduction and fixation of the anterior pelvic ring. Worse still, the posterior approach should not be performed for patients with Morel–Lavellee injury in case of deep infection.

In recent years, the LRA has achieved good results in the treatment of posterior pelvic ring fracture combined with nerve injury,^24^, ^34^ for it can expose the hemi‐pelvic ring extra‐peritoneally. To reveal the external oblique aponeurosis, make an incision through the skin, Camper fascia, and Scarpa fascia. Cut the external oblique, internal oblique, and the transverse fascia abdominis to access the extraperitoneal space, which is located above the inguinal ligament and to the inside of the spermatic cord or round ligament of the uterus and the outside of the rectus abdominis muscle and inferior abdominal artery. Attention should be given throughout the separation process to avoid damaging the peritoneum and to protect the vas deferens, ureters, and blood vessels located underneath the abdominal wall.

The middle window of the LRA can be exposed by pulling the external iliac vascular bundle, spermatic cord or round ligament medially, and the iliopsoas laterally. The sacroiliac joint, the large ischial notch, the upper acetabulum, and the obturator nerve can all be seen through this window. The medial window is exposed by pulling the iliac vascular bundle and spermatic cord or round ligament of uterus outwardly as the outside boundary, and gently pressing and pulling the peritoneum and contents inwardly, through which surgeons can reduce and fix the fractures of the pubic symphysis, suprapubic ramus, and anterior acetabular column. The lateral window can expose the internal side of the entire ilium by pulling the ilium muscle outward and the psoas major muscle inward in the space between these two muscles. Fractures of the ilium can be reduced and fixed through this window.

The middle window can easily reveal the periphery of the sacroiliac joint, which can expose and protect the internal and external iliac vessels and the lumbosacral plexus under direct vision. At the same time, the anterior dislocation of the sacroiliac joint can be unclenched and repositioned. Especially for patients with irreducible ADSIJ, it is easier to perform osteotomy on the auricular surface of the ilium through the middle window of the LRA. The surgical risk and reductive difficulty of the LRA are less than that of the ilioinguinal approach for its better exposure and less iatrogenic injury. All 15 patients in this group reduced and fixed the ADSIJ *via* the LRA and three of them underwent iliac osteotomy treatment due to difficult reduction, which had good clinical efficacy at the follow‐up. The intraoperative blood loss and surgical time were less than that of traditional surgical treatment of posterior pelvic ring fracture.^24^, ^34^ To sum up, the LRA in the treatment of ADSIJ is feasible.

### Evaluation of Clinical Efficacy

ADSIJ injuries have clear surgical indications. Nonoperative treatment often leads to malunion or nonunion of the pelvic ring, seriously affecting the quality of patients' life. The rehabilitation of limb function depends on multiple factors, including the severity of the original injury and the reduction quality of fracture dislocation. Iatrogenic injury and complications are also important factors affecting limb function. As lack of reports on large cases to evaluate the clinical efficacy of ADSIJ, it is difficult to compare with other literature. Most of the ADSIJ injuries are associated with other parts of the pelvic ring lesions, making it difficult to record the operation time and the amount of intraoperative bleeding. In our group, the operation time and the intraoperative blood loss of the LRA for ADSIJ are less than those of other approaches for posterior pelvic ring injury reported in the literature,^24^, ^34^ which can indicate that the reduction and fixation of ADSIJ by the LRA will not increase the operation time and intraoperative bleeding. Among 15 patients in this group, the excellent and good rates of reduction and limb functional recovery 1 year after surgery were 80% and 73.3%, respectively. Three patients in this group had complete dislocation of the sacroiliac joint, and all achieved anatomical reduction, with well recovery of the limb function at the 1‐year follow‐up examination after surgery (the Majeed function score of 90 points). Two patients with old ADSIJ had general reduction quality of fracture and dislocation, and their recovery of limb function was poor at the 1‐year postoperative follow‐up. All in all, the LRA can achieve a satisfactory reduction and good clinical efficacy for fresh ADSIJ, while poor reduction and effect for the old one. The ADSIJ has a high rate of lumbosacral plexus injury. The LRA can decompress and release the lumbosacral plexus while reducing the anterior dislocation, which can achieve better neurological recovery. In this group, all eight patients combined with lumbosacral plexus injury suffered neurolysis. The recovery rate of neurological function was 87.5% (7/8) at the 1‐year follow‐up, which was higher than that reported in the literature.^24^ This indicated that it is feasible and effective to perform lumbosacral plexus decompression during the process of fracture reduction *via* the LRA in ADSIJ patients with lumbosacral plexus injury.

### Strengths and Limitations

Comparing with the existing anterior approaches, the LRA is a feasible and safe option for fixing ADSIJ injury. Our study has provided a new surgical option to treat ADSIJ injury, which is even suitable for those with lumbosacral plexus injury. However, there are several limitations, including its single‐center retrospective nature, limited number of patients and short‐time follow‐up. Hence, a larger number of patients should be included, and a longer follow‐up needs to be incorporated in future studies.

### Conclusion

The ADSIJ injury is a special type of pelvic ring injury, manifesting severe damage to the integrity and stability of the pelvic ring. It can achieve satisfactory reduction and fixation of the ADSIJ injury by the LRA. For ADSIJ patients with lumbosacral plexus injury, the LRA can help effectively restore nerve function by releasing the lumbosacral plexus while reducing fractures and dislocation.

## Author Contributions

Shicai Fan and Sheqiang Chen share first authorship. Shicai Fan: conceptualization, methodology, writing—original draft, funding acquisition. Sheqiang Chen: data curation, writing—original draft, writing—review and editing. Qiguang Mai, Tao Li: validation. Yuhui Chen, Zhenhua Zhu: investigation, resources. Hua Wang, Cheng Yang: data curation. Jianwen Liao, Ruipeng Zhang: writing—review and editing. Yingze Zhang: conceptualization, project administration.

## Funding Information

This study was supported by the National Natural Science Foundation of China (82072411), Innovation fund cultivation project of National Clinical Research Center for Orthopedics Sports Medicine and Rehabilitation (2021‐NCRC‐CXJJ‐PY‐06). National Key Research and Development Program of China (2022YFC2504303).

## Conflict of Interest Statement

The authors have declared that there are no competing interests.
